# Reparameterized multiobjective control of BCG immunotherapy

**DOI:** 10.1038/s41598-023-47406-z

**Published:** 2023-11-27

**Authors:** Rongting Yue, Abhishek Dutta

**Affiliations:** Electrical and Computer Engineering, University of Connecticut, Storrs, 06269 USA

**Keywords:** Immunotherapy, Electrical and electronic engineering

## Abstract

Bladder cancer is a cancerous disease that mainly affects elder men and women. The immunotherapy that uses Bacillus of Calmette and Guerin (BCG) effectively treats bladder cancer by stimulating the immune response of patients. The therapeutic performance of BCG relies on drug dosing, and the design of an optimal BCG regimen is an open question. In this study, we propose the reparameterized multiobjective control (RMC) approach for seeking an optimal drug dosing regimen and apply it to the design of BCG treatment. This approach utilizes constrained optimization based on a nonlinear bladder cancer model with impulsive drug instillation. We compare the performance of RMC with Koopman model predictive control (MPC) and validate the efficacy of optimal BCG dosing regimens through numerical simulations, demonstrating the efficient elimination of cancerous cells. The proposed control framework holds the potential for generalization to other model-based treatment designs.

## Introduction

Cancer is a significant global public health concern and a leading cause of mortality. Bladder cancer, a subtype of cancer, accounts for approximately 7.6% of cancer-related deaths in men and 2.2% in women, based on the 5-year average annual percent change (2015)^1^. The high mortality rate associated with bladder cancer necessitates the development of highly effective treatment regimens. Chemotherapy and immunotherapy are two commonly recommended approaches for different stages of bladder cancer due to their efficacy^2,3^. While chemotherapy directly targets and kills cancer cells, immunotherapy enhances the body’s immune system to combat neoplastic diseases. One widely used immunotherapy for early-stage bladder cancer is Bacillus Calmette–Guérin (BCG). BCG is highly effective, with a success rate of approximately 90%. It involves the instillation of live BCG into the affected area to activate lymphocytes and stimulate an antitumor response from the immune system^4^. The regulation of T lymphocytes, such as CD4+ and CD8+, plays a crucial role in cancer immunotherapy by directing antigen-specific cytotoxicity^5^. Following instillation, BCG attaches to urothelial cells and is internalized by bladder cancer cells. The cancer cells release cytokines and regulate molecules such as MHC II and ICAM-1 to present processed antigens, thereby activating cytotoxic T-cells. This immune response mediates the cytotoxicity of immune cells, leading to the elimination of cancer cells^4^.

BCG treatment regimens have been studied for decades. Though BCG is therapeutically effective for bladder cancer, the dosing regimen of BCG is dedicated and needs to be designed carefully to ensure its efficacy. On the one hand, high doses of drugs result in the fast elimination of cancerous cells, while high drug toxicity and severe side effects may cause undesired harmful effects to patients. On the other hand, low drug doses will result in a great loss in therapeutic efficacy. In bladder cancer treatment, a high dose of BCG could lower the efficacy of cytokines induction and lead to the termination of treatment due to severe side effects, including BCG sepsis^6^. The clinical characteristics of different BCG can be found in a literature search^7^. Thus, there are constraints on drug doses due to patients’ health conditions, and how to design the optimal doses and duration for patients in different cancerous stages is an open question. Designing optimal dosing regimens for BCG treatment is challenging due to constraints and nonlinearity in the treatment model.

Control theory is essential for effective disease management strategies, ensuring precise control of treatment interventions. Control theory focuses on dynamic systems and aims to regulate their behavior to align the system’s state with a desired reference^8^. A control system employs sensors for system state measurement, actuators for control inputs, and a controller to compute these inputs based on feedback and reference, ensuring desired outcomes are achieved^9^. This incorporation of measurements enables precise regulation of system dynamics. In systems pharmacology, nonlinear dynamics govern the intricate relationships between drug dosages, patient responses, and disease progression, which can be captured by nonlinear dynamic models^10^. Measurement-driven feedback control adapts in real-time to system variations, enhancing the precise control of disease treatment. Nevertheless, achieving optimal treatment outcomes is contingent on devising optimal dosing regimens based on nonlinear drug response and disease progression.

Various control strategies are available for determining dosing schemes for disease treatments. Feedback control has been pivotal in diverse contexts, from stabilizing the COVID-19 pandemic through vaccination strategies^11^, to precise management of neuromuscular blocking agent concentrations during anesthesia via Proportional-Integral-Derivative (PID) control^12^. Lyapunov stability-based controllers can be used to stabilize COVID-19 infections by controlling the number of tests in regulating quarantine strategies^13^. Optimal control strategies like the Linear Quadratic Regulator have found application in linear systems, albeit with a need for preliminary linearization when dealing with nonlinear models^14^. Multi-objective optimal control has been used to optimize drug rescheduling, balancing doses, efficacy, and toxicities as seen in the case of remdesivir^15^. Model predictive control (MPC) has been used for addressing constraints in drug dosing optimization problems, such as optimizing chemotherapy dosing in cancer treatment^16^. A comprehensive exploration of diverse drug dosing strategies is available in^10^.

MPC predicts state variable changes in a plant model and iteratively calculates control laws using finite-horizon optimization^17^. While MPC can handle nonlinear control problems, the nonlinearity in the dynamic model introduces challenges in terms of non-convex optimization and convergence/stability of the algorithm^18^. Nonlinear MPC works directly on nonlinear models, but a quadratic positive semi-definite stage cost is necessary to ensure closed-loop stability^19^. Linearization techniques are usually applied to obtain linear models. The Koopman operator has gained attention for its ability to approximate nonlinear models effectively^20–22^. By approximating the nonlinear dynamics of the original plant model using the linear evolution of observables, the Koopman operator transforms the non-convex optimization problem in MPC for a nonlinear model into a convex quadratic programming problem^23^.

Besides, the optimal dosing schemes can be designed by reparameterizing the control process, which handles nonlinear relationships between variables in the model^24^. Also, reparameterization allows simultaneous optimization of multiple control objectives in control design by effectively incorporating various performance criteria, cost functions, or constraints^25,26^. This capability facilitates trade-off analysis and the design of control strategies that achieve a balance between conflicting objectives. Additionally, a reparameterized control framework allows the control strategies to be generalized to similar control problems with minimal modifications, streamlining the control design process and enhancing scalability. In this study, we propose the reparameterized multiobjective control (RMC) as a constrained open-loop optimal control strategy and apply it to the BCG treatment model for optimizing the dosing scheme. RMC provides a framework for optimal impulsive dosing strategies for the nonlinear treatment models with constraints. We also use Koopman MPC to compare the drug dosing scheme.

## Materials and methods

### Nonlinear bladder cancer model

**Figure 1:**
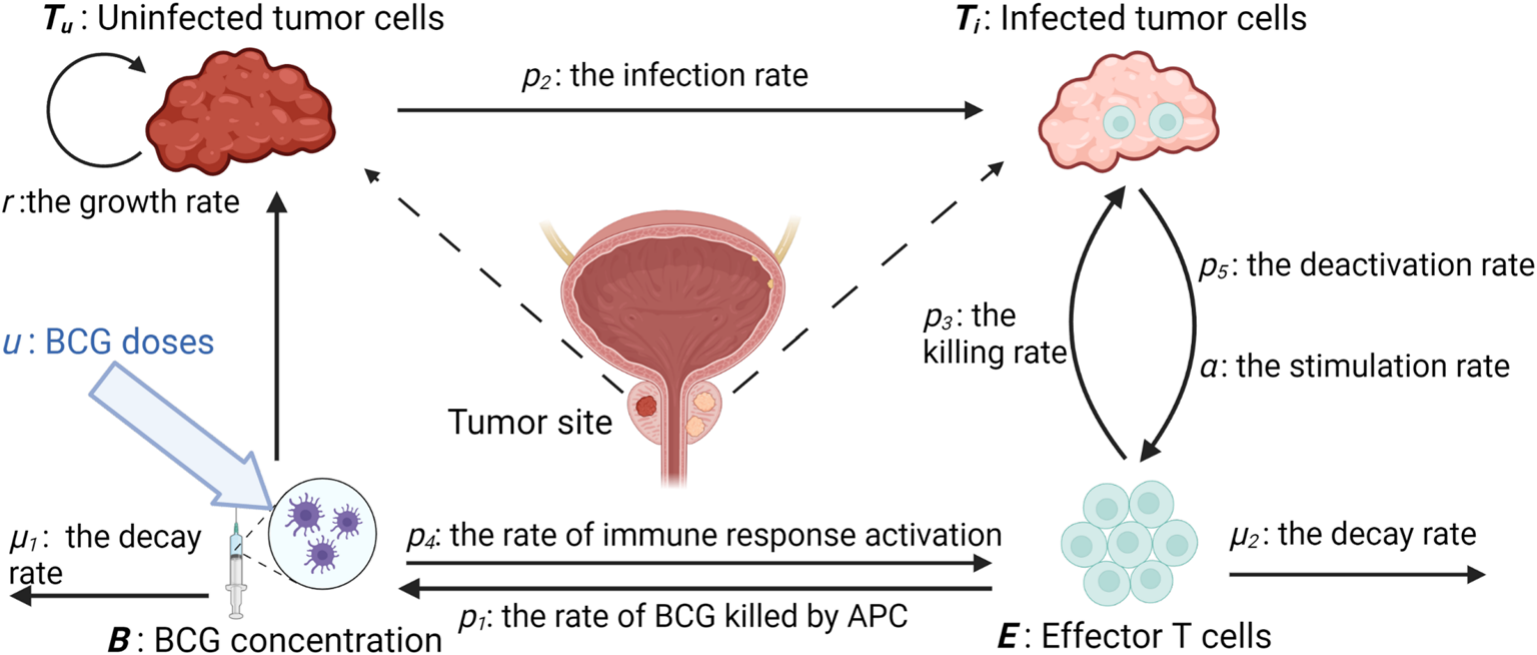
BCG treatment model for bladder cancer. BCG is instilled into tumor sites to bring uninfected tumor cells $T_u$ to infected tumor cells $T_i$ that will be eliminated by effector T cells *E*. Figure is adapted from^27^ and is created with BioRender.com.

In this study, a bladder cancer model^27,28^ is employed, as depicted in Fig. 1. The model considers the impact of BCG vaccines on various populations, including uninfected tumor cells, infected tumor cells, and effector T cells. The administration of BCG vaccines to the tumor site converts uninfected tumor cells into infected tumor cells, which are subsequently targeted and eliminated by the effector cells.

Consider a general dynamic system with impulsive input:

1 $$\begin{aligned} \begin{aligned} {\dot{X}}(t)&= f_X(X(t)), \quad t \ne \tau _k\\ X(t^+&) = X(t) + B_{in}u_{in}(t), \quad t =\tau _k, \quad k = 1, 2,... \end{aligned} \end{aligned}$$

where $X(t) \in {\mathbb {R}}^{n_s}$ is the state vector that contains $n_s$ state variables, $f_X: {\mathbb {R}}^{n_s} \rightarrow {\mathbb {R}}^{n_s}$ is a function of dynamics of *X*(*t*), *t* is the time index and $t^+$ refers to the time instant after *t*, $B_{in} \in {\mathbb {R}}^{n_s}$ is the input coefficient vector, $u_{in}(t)$ is the input, and $\tau _k$ ($k = 1, 2,...$) is the time instant that the impulsive input is applied to the model. We use the BCG treatment model in the form of nonlinear differential equations (in Eq. 2 from^27^ with impulsive inputs) that govern the concentrations of four states: BCG concentration (*B*) measured in units of $1\times 10^6$ colony-forming units (c.f.u), activated immune system cell population (*E*) measured in units of $1\times 10^6$ cells, infected tumor cell population ($T_i$) measured in units of $1\times 10^6$ cells, and uninfected tumor cell population ($T_u$) measured in units of $1\times 10^6$ cells.

2 $$\begin{aligned} \begin{aligned} {{\dot{B}}(t)}&{= -\mu _1B(t)-p_1E(t)B(t) - p_2B(t)T_u(t)}\\ {\dot{E}}(t)&= -\mu _2E(t)+\alpha T_i(t)+p_4E(t)B(t)-p_5E(t)T_i(t)\\ {\dot{T}}_i(t)&= -p_3E(t)T_i(t)+p_2B(t)T_u(t)\\ {\dot{T}}_u(t)&= -p_2B(t)T_u(t)+r(1-\beta T_u(t))T_u(t) \end{aligned} \end{aligned}$$

where $\mu _1$ and $\mu _2$ are the decay rates of BCG and effector cells, respectively. $p_1-p_5$ are the rates of transitions between cells. $\alpha$ is the infected tumor stimulation rate. $\beta$ is the inverse of tumor carrying capacity. *r* is the tumor growth rate and *u* is the BCG dose. BCG is instilled into the tumor site, and optimal scheduling of dose *u*(*t*) is measured in units of $1\times 10^6$ colony-forming units (c.f.u), is to be designed to eliminate $T_u$. Then we have $X(t) = [B(t), E(t), T_i(t), T_u(t)]^T$ and $B_{in} = [1, 0, 0, 0]$ based on (1). Specifically, we have $B(t^+) = B(t) + u(t)$ for $t = \tau _k$ ($k=1,2,...$), where $\tau _k$ is the time instant that BCG is given. The description and numerical values of the parameters can be found in Table 1^27,28^.

**Table 1:** List of all parameters in the BCG treatment model^27,28^.

Parameter	Description	Units	Source value	Dimensionless estimate	Source
$\mu _1$	The rate of BCG decay	$day^{-1}$	0.1	1	^29^
$\mu _2$	The rate of effector cells decay	$day^{-1}$	0.041	0.41	^30,31^
$p_1$	The rate of BCG killed by APC	$cell^{-1}day^{-1}$	$1.25\times 10^{-7}$	1.25	^32,33^
$p_2$	Infection rate of tumor cells by BCG	$cell^{-1}day^{-1}$	$0.285\times 10^{-7}$	0.285	^34,35^
$p_3$	Destruction rate by effector cells	$cell^{-1}day^{-1}$	$1.1\times 10^{-7}$	1.1	^30,31^
$p_4$	Immune response activation rate	$cell^{-1}day^{-1}$	$0.12\times 10^{-7}$	0.12	Not found
$p_5$	Effector cells deactivation rate	$cell^{-1}day^{-1}$	$0.345\times 10^{-9}$	0.003	^30,31^
$\alpha$	Infected tumor stimulation rate	$day^{-1}$	0.052	0.52	^32^
$\beta$	Inverse of tumor carrying capacity	$cell^{-1}$	$1.1\times 10^{-8}$	0.011	^36^
*r*	Tumor growth rate	$day^{-1}$	0.0032	0.032	^37^

The origins and sources of these parameters are referenced from^28^ and are elaborated as follows: $\mu _1$ was estimated through experimental work involving the cultivation of the Mycobacterium avium strain based on the experimental data in^29^; *r* was derived from a median growth rate determined through an in vivo study^37^, where a logistic model for tumor growth was fitted to mammographic measurements from breast cancer patients using the least squares solution; $\beta$ was computed under the assumption of a cylindrical tumor shape with a depth of 3 cell layers. A maximal diameter of 6.4 cm (reported by^36^) is employed in the calculations; $\mu _2$, $p_3$, and $p_5$ were estimated in^30^ by fitting experimental data of chimeric mice with Bcl-1 lymphoma in their spleen^31^ in the sense of nonlinear least squares, using the Hooke–Jeeves optimization method; $p_1$ was obtained in^32^ as the max killing rate of bacteria by activated macrophages that can serve as Antigen Presenting Cells, based on experiments on Mycobacterium bovis BCG Substrains in^33^; $\alpha$ was characterized as the maximum Th1/Th2 cell recruitment rate in^32^ based on experiments about lymphocyte recruitment facilitated by Interleukin-8 in the inflammatory process in^38^; $p_2$ was informed by^34^ and^35^ based on the internalization of BCG by incubating human bladder cancerous cell lines like T24^34^ and the adhesion and internalization of BCG and effector cells^35^. Furthermore, $p_2$ and its numerical values are also reported in^39^ and supported by clinical trial data^40^. The source for $p_4$ is not identified as informed by^28^. However, sensitivity analysis showed that $p_4$ minimally influences the output settling time, which will be shown later.

The model is discretized using the fourth order-Runge Kutta method, and the resulting discrete model has a state vector $X(k) = [ B(k), E(k), T_i(k), T_u(k)]^T$ at time *k* with the output $y_k = y(k) = T_u(k)$ and input *u*(*k*). The objective of this study is to optimize the scheduling of the dose *u* to eliminate the uninfected tumor cells $T_u$. The BCG vaccine is administered at the tumor site using the dose *u*. The observed output of the immunotherapy model is assumed to be the population of uninfected tumor cells $T_u$. If the system is observable, estimators can be used to obtain the values of other states. Based on guidelines from the Food and Drug Administration, each vial of the BCG vaccine contains 1 to 8 $\times 10^8$ c.f.u, which is approximately 50 mg (wet weight). A previous study on BCG doses^7^ suggests that the weekly dose ranges from 40 to 120 mg. Standard doses are typically 80 or 120 mg, which have shown better efficacy than lower doses (one-third to two-thirds of the standard dose). The treatment duration varies from 6 to 12 weeks. The scaling of the cell population is $1\times 10^6$ cells for the dimensionless cell population values.

### Reparameterized multiobjective control

**Figure 2:**
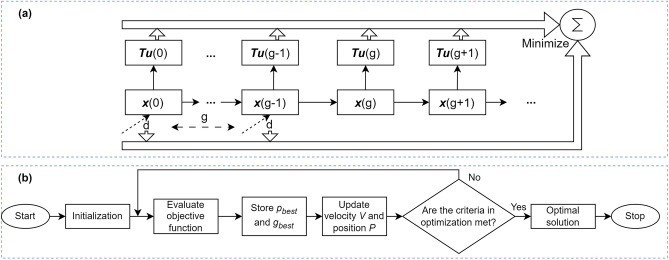
(**a**) Diagram of the objective function in RMC. The multiobjective control designs the drug dosing scheme that minimizes the cumulative uninfected tumor cell population and the cumulative drug dosages. *d* is the dosage of a single treatment, and *g* is the gap between two consecutive treatments. ${\textbf {x}}(g)$ is the state vector that contains uninfected tumor cell population $T_u(g)$ at time *g*. (**b**) The flowchart of PSO algorithm (adapted from^41^). $p_{best}$ and $g_{best}$ are the individual and global optimum values of the particles.

Denote the sampling time as $\Delta t$ and a unit dose of BCG as *d* (unit: 1 $\times 10^6$ c.f.u). The time interval (or gap) between two consecutive doses is *g* (unit: days). The total number of treatments for the dosing scheme is *N*. Then the cumulative drug doses can be formulated as $d \cdot N$. The cumulative uninfected tumor cell population is $\sum _{k=0}^{k_t} T_u (k) \cdot \Delta t$, which is in the form of the area of the uninfected tumor cell population across time. The control objective is to design an optimal BCG dosing scheme to eliminate tumor cells when considering BCG cost. Thus, the objective function to be minimized comprises the cumulative population of uninfected tumor cells and cumulative BCG vaccines whose diagram is visualized in Fig. 2(a).

The terminal constraint on $T_u(k_t)$ is set to tumor-free condition $T_u = 0$ at the end of the treatment (i.e., $k = k_t$), which is fixed as the maximal treatment period, i.e., $k_t =max(g) \cdot max(N)= 100$. This constraint is relaxed with penalty weight $w_t$. Besides, as the number of treatments has to be a positive integer within the boundary, a hard constraint $N \in {\mathbb {Z}}^+$ is applied. To enhance the constraint’s flexibility, we introduce relaxation through penalty terms in the objective function, controlled by penalty parameters. Specifically, we add the residual $N - \text {round}(N)$ as a penalty term weighted by $w_N$ in the cost function. Furthermore, in the cost function, we normalize $T_u$ in both the stage cost and terminal cost using the maximal $T_u$ within the 100-day time horizon in the absence of BCG vaccines (i.e., $u = 0$).

The objective function is shown as follows

3 $$\begin{array}{*{20}c} {\min _{{\{ d,N,g\} }} \left( {\sum\limits_{{k = 0}}^{{k_{t} }} {T_{u} } (k) \cdot \Delta t} \right) \cdot w_{1} + \lambda \cdot d \cdot N + w_{N} |N - {\text{round}}(N)| + w_{t} \cdot T_{u} \left( {k_{t} } \right)} \hfill \\ {{\text{subject}}\;{\text{to:}}\;{\mathbf{x}}(k + 1) = f({\mathbf{x}}(k),u(k))} \hfill \\ {u(k) = \sum\limits_{{n = 0}}^{{N - 1}} d \cdot [s(k - ng/\Delta t) - s(k - ng/\Delta t - 1)]} \hfill \\ {2.2 \le d \le 6.4} \hfill \\ {5 \le g \le 10} \hfill \\ {3 \le N \le 10} \hfill \\ {T_{u} (k_{t} ) = T_{{k_{t} }} } \hfill \\ \end{array}$$

where *s*(*k*) is a step function of time index *k* which is used to shape the input signals, as BCG vaccines are given to patients impulsively. The nonlinear model process $f({\textbf {x}}(k),u(k))$ is the function of state vector ${\textbf {x}}(k)$ (which contains $T_u(k)$) and input *u*(*k*). The penalty weight, denoted as $\lambda$, is employed to fine-tune the relative significance of two factors: cumulative tumor cells and drug concentration. A higher value of $\lambda$ signifies a greater emphasis on administering fewer BCG vaccines, thereby permitting more cancerous cells to persist in patients throughout the treatment process. On the other hand, a lower value of $\lambda$ prompts the controller to prioritize tumor elimination, even if it requires a relatively larger quantity of BCG vaccines to achieve this objective. As proposed by^28^, the dosage constraint applies to a single treatment. The parameter *g* is derived from the current weekly dosing scheme of BCG treatment, as outlined by the FDA. To provide a more flexible timeframe, the range of *g* has been slightly expanded from 5 to 10 days. Moreover, based on FDA guidelines, a weekly treatment scheme typically comprises a total of six treatments, spanning over 42 days. To accommodate this expanded range, we define the period as ranging from $min(g)\cdot min(N)$ to $max(g)\cdot max(N)$.

The optimal parameter set that minimizes the objective function is to be searched by optimization algorithms. We first employ Particle Swarm Optimization (PSO)^42^. PSO iteratively updates the position $P^i(k) \in {\mathbb {R}}^3$ and velocity $V^i(k) \in {\mathbb {R}}^3$ of each particle *i* using the following update equations:

4 $$\begin{aligned} P^i(k+1)= & {} P^i(k) + V^i(k+1) \end{aligned}$$

5 $$\begin{aligned} V^i(k+1)= & {} w V^i(k) + c_1 r_1 (p_{best}^i - P^i(k)) + c_2 r_2 (g_{best} - P^i(k)) \end{aligned}$$

where the inertia weight constant $w \in [0,1]$ retains a portion of the particle’s previous search direction and speed. The constants $c_1$ and $c_2$ assign weights to the impact of individual particles and all particles, respectively. The random numbers $r_1$ and $r_2$ are sampled from a standard uniform distribution *U*[0, 1] and help prevent premature convergence. $p_{best}^i$ is the best position of particle *i*, and $g_{best}$ is the best position of all particles. The term $(p_{best}^i - P^i(k))$ guides the particle back to its best position encountered so far, while $(g_{best} - P^i(k))$ brings the particle back towards the overall best position found among all particles. The flowchart of PSO is visualized in Fig. 2(b)^41^.

In addition, we employ two other metaheuristic optimization techniques (Ant Colony Optimization (ACO)^43^ and Simulated Annealing^44^) to compare their cost-associated optimal solutions to PSO using the same initial point and evaluate changes in the minimum cost function value and optimal dosing schemes. Simulated annealing probabilistically explores solution spaces by making random moves, guided by a probability parameter *P*^44^. Denote $\Delta c$ as the difference in cost between the new and old solutions. If a move reduces cost ($\Delta c < 0$), it’s accepted with certainty ($P = 1$), while if it increases cost ($\Delta c > 0$), it’s accepted with a decreasing probability ($P = e^{-\frac{\Delta c}{T}}$). The temperature *T* decreases geometrically over time ($T(t+1) = T(t) \cdot C_r$). In ACO algorithm, Ant *k* selects an edge connecting vertices *x* and *y* using probability $p^k_{xy}$ defined as $\frac{(\rho ^{\alpha }_{xy})(\eta ^{\beta }_{xy})}{\sum _{z\in allowed_y} (\rho ^{\alpha }_{xy})(\eta ^{\beta }_{xy})}$. Here, $\rho _{xy}$ represents pheromone on edge *xy*, and $\eta _{xy}$ indicates move attractiveness, with $\alpha$ and $\beta$ controlling their influence. Pheromone is updated as $\rho _{xy} \leftarrow (1-\epsilon )\rho _{xy} + \sum _k^m \Delta \rho ^k_{xy}$, where $\epsilon$ is pheromone evaporation rate, *m* is the total ants, and $\Delta \rho ^k_{xy}$ is pheromone deposited by ant *k*, equal to $Q/L_k$ if it traverses edge *xy*.

### Koopman model predictive control

We employ the Koopman MPC method^23^ on the BCG treatment model, incorporating impulsive control inputs. This approach, previously implemented in our study^45^, is utilized to design an optimal dosing scheme and serves as a basis of comparison against RMC. To capture the model’s nonlinear behaviors, we employ Koopman linearization^46^. This method utilizes a finite subset of the infinite-dimensional Koopman operator ($\kappa$), mapping observable variables into a higher-dimensional space with new basis functions. This enables a linear transformation among observables^46,47^.

**Figure 3:**
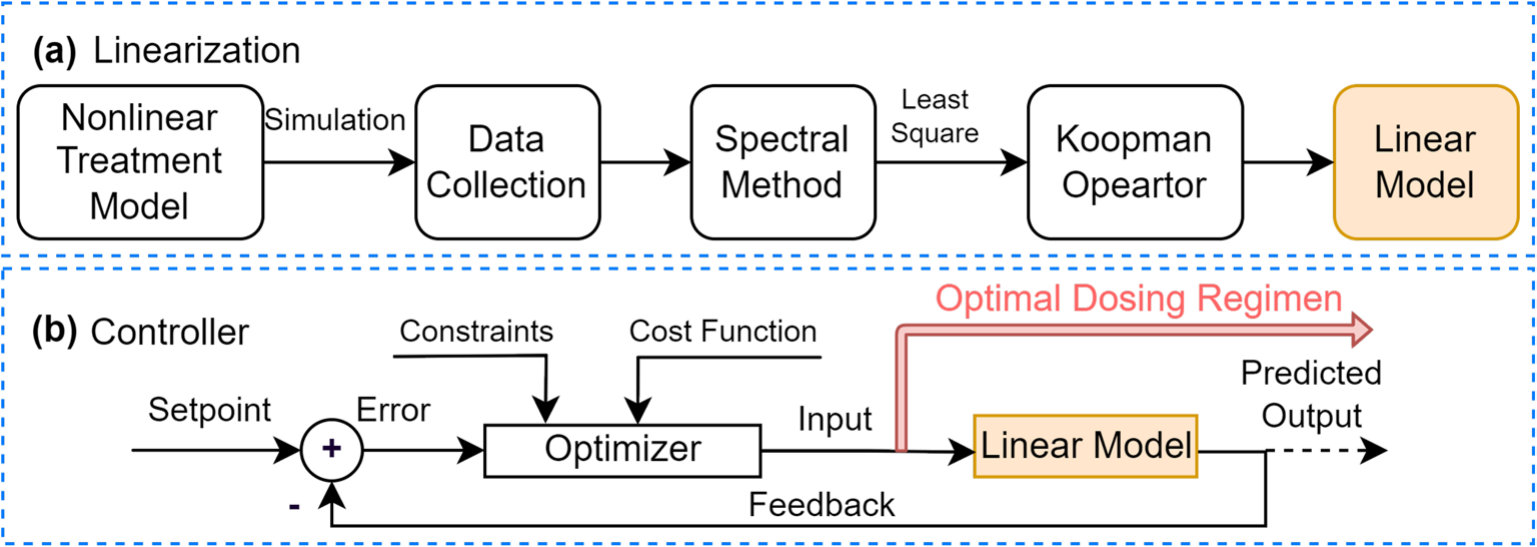
The design of optimal drug dosing scheme using Koopman MPC. (**a**) Koopman linearization process. The Koopman operator utilizes spectral methods to transform the nonlinear model into a linear one. This process involves using time series data generated from the nonlinear model. (**b**) Linear MPC algorithm based on the linearized model. Constrained optimization is performed to obtain the optimal control sequence, which corresponds to the dosing scheme. This optimized sequence will then be implemented on the original nonlinear system. Figure adapted from our previous study^45^.

The extended state $Z_k \in {\mathbb {R}}^{N}$ is formed by combining observables and control inputs. Its dimensionality is given by $N = n_y(n_d+2) + (n_d+1)n_u$^23^, where $n_d$ is the number of delay units, $n_y$ (set as 1) is the number of outputs, and $n_u$ is the number of inputs. With time series data spanning $y_k$ to $y_{k+n_t}$ for outputs and $u_k$ to $u_{k+n_t-1}$ for inputs over a total simulation time $n_t$, the extended state at time step *k* is expressed as $Z_k = [y_{k+n_t-1},\dots ,y_k, u_{k+n_t-2},\dots ,u_k]^T$. The one-step-ahead extended state is denoted as $Z_{k+1} = [y_{k+n_t},\dots ,y_{k+1}, u_{k+n_t-1},\dots ,u_{k+1}]^T$. The dynamics of *Z*(*k*) are described by the Koopman operator $\kappa$: $(\kappa \phi )(Z_{k}) = \phi (f(Z_{k}))$. Here, the function $f: {\mathbb {R}}^N \rightarrow {\mathbb {R}}^N$ captures the dynamic behaviors of $Z_k$, and $\phi : {\mathbb {R}}^N \rightarrow {\mathbb {K}}$ maps the lifted function space to Koopman space. In the infinite-dimensional space, the Koopman operator has a spectrum $\kappa \phi = \sum ^\infty _{i=1} \lambda _i m_i e_i$, where $\lambda _i$ represents Koopman eigenvalues, $m_i \in {\mathbb {K}}$ are weights, and $e_i$ are Koopman eigenfunctions^48^. We use Extended Dynamic Mode Decomposition^48^ to approximate the Koopman operator. This involves lifting system observables over Radial Basis Functions (RBFs) to enhance accuracy. Specifically, thin plate splines RBFs $B(Z_k,C) \in {\mathbb {R}}^{N_{rbf}}$ are used as suggested in^48^ due to the irregular nature of the Koopman spectral analysis, where $N_{rbf}$ represents the number of RBFs. The lifted state $Z_{lift}(k) \in {\mathbb {R}}^{(N + N_{rbf})}$ is obtained by stacking $Z_k$ and $B(Z_k,C)$ as follows: $Z_{lift}(k)=[Z_k, B(Z_k, C)]^T$. The linear evolution from $Z_{lift}(k)$ to $Z_{lift}(k+1)$ with input *U*(*k*) is obtained, given by $Z_{lift}(k+1) = A_{lift} Z_{lift}(k) + B_{lift} U(k)$, where $A_{lift}$ is the state transition matrix ($A_{lift} \in {\mathbb {R}}^{(N + N_{rbf})\times (N + N_{rbf})}$) and $B_{lift}$ is the input matrix ($B_{lift}\in {\mathbb {R}}^{(N + N_{rbf})\times n_u}$). The Frobenius norm of the difference between the observables and the states generated by the finite-dimensional Koopman-linearized model is minimized using the least-squares solution, i.e., $\parallel (\kappa \phi )(Z_{lift}(k)) - [A_{lift}, B_{lift}] [Z_{lift}(k), U(k)]^T\parallel _F$, where $\parallel \cdot \parallel _{F}$ denotes the Frobenius norm. Furthermore, a projection from the Koopman space to the original space is desired. This is accomplished by using $C_{lift} \in {\mathbb {R}}^{(N + N_{rbf})}$ to predict ${\hat{X}}(k)$ in the function space of *X*(*k*) from the function space of $Z_{lift}(k)$, where ${\hat{X}}(k) = C_{lift} Z_{lift}(k)$. The matrix $C_{lift}$ is obtained by minimizing $\parallel X(k) - C_{lift} Z_{lift}(k) \parallel _F$. By stacking $Z_{lift}(k)$ and *U*(*k*), the least-squares solution is obtained as shown in Eq. (6).

6 $$\begin{aligned} { \begin{bmatrix} A_{lift} &{}\quad B_{lift} \\ C_{lift} &{}\quad 0 \end{bmatrix} = \begin{bmatrix} Z_{lift}(k+1)\\ X(k) \end{bmatrix} \begin{bmatrix} Z_{lift}(k)\\ U(k) \end{bmatrix}^\dagger } \end{aligned}$$

where $\dagger$ is pseudo-inverse operatoration. Time series data comprising observables and inputs are obtained by simulating the model using random initial states and input sequences that satisfy the specified constraints. The process of Koopman linearization is summarized in Fig. 3a. Next, we adopt the MPC algorithm^17^ for the design. We use notations $A_c = A_{lift}$, $B_c = B_{lift}$, and $C_c = C_{lift}$ from Eq. (6) and denote the initial condition $X(k) = X_k$. The optimization problem in MPC at time *k* can be formulated as follows

7 $$\begin{aligned} { \begin{aligned} \min _{{\textbf {u(k)}}} \sum _{i=0}^{H_p-1} \parallel X (k+i) -&X_r(k+i) \parallel _Q^2 + \sum _{j=0}^{H_u - 1} \parallel u (k+j)\parallel _R^2 + \parallel X(k+H_p) \parallel ^2_P\\ s.t. \,&Z_{lift}(k+1) = A_c Z_{lift}(k) + B_c u(k)\\&X(k+i) = C_c Z_{lift}(k+i)\\&u_{min} \le u(k) \le u_{max}\\&X(k) = X_0\\&X(k_t) = X_{k_t}\\&i = 0, 
\dots , H_p-1\\&j = 0, \dots , H_u - 1 \end{aligned}} \end{aligned}$$

where $y_r(k+j) \in {\mathbb {R}}$ is the set point on reference trajectory $y_r(k+j) = T_u(0) e^{-(k+i)\cdot \Delta t}$ that decays exponentially from its initial value to the terminal value $X_{k_t}$ after 6 weeks. We only use the terminal constraint on $T_u$. The control input sequence at time *k*, denoted as ${\textbf {u(k)}} : = \{u(k+j)\}_{j=0}^{H_p-1}$, represents control actions over a prediction horizon. Control inputs beyond the control horizon ($H_u \le j\le H_p -1$) are set to zero. A single treatment’s minimum and maximum dosage limits are denoted as $u_{min}$ and $u_{max}$, respectively. The initial condition is $X_0$. Weights $Q\in {\mathbb {R}}^{4\times 4}$ and $R\in {\mathbb {R}}$ determine the penalties for state deviation and control effort. *Q* and *R* are positive semidefinite ($Q, R \succeq 0$). $H_p$ is the prediction horizon and $H_u$ is the control horizon. The $l_2$ norm ($\parallel \cdot \parallel$) measures state deviation and control effort. The diagonal weight matrix $P \in {\mathbb {R}}^{4 \times 4}$ represents the terminal cost weighting and is positive semidefinite ($P \succeq 0$). *P* is obtained from a discrete-time algebraic Riccati equation $P = A_c^T P A - (A_c^T P B_c)(B_c^T P B_c + R)^{-1}(B_c^T P A_c) + Q$. Let $X(k)^{ } = [x(k+1)^{ }, x(k+2)^{ }, \dots , x(k+H_p)^{ }]^T$, $U(k)^{ } = [u(k)^{ }, u(k+1)^{ }, \dots , u(k+H_u-1)^{ }, 0, \dots , 0]^T$, and $W = [A_c^{ }, A_c^2, \dots ,A_c^{k+H_p}]^T$. The state $x(k+i)$ can be expressed as $x(k+i) = A_c^i x(k) + G_i U(k)$, where *i* denotes the time index, and $G_i$ represents the *i*-th row of $$G = \left( {\begin{array}{*{20}l} {B_{c} } \hfill & 0 \hfill & \ldots \hfill & 0 \hfill \\ {A_{c} B_{c} } \hfill & {B_{c} } \hfill & \ldots \hfill & 0 \hfill \\ \ldots \hfill & \ldots \hfill & \ldots \hfill & \ldots \hfill \\ {A_{c}^{{H_{p} - 1}} B_{c} } \hfill & {A_{c}^{{H_{p} - 2}} B_{c} } \hfill & \ldots \hfill & {B_{c} } \hfill \\ \end{array} } \right)$$.

Due to the impulsive inputs, only the first element in the control sequence *U*(*k*) is nonzero, resulting in all zero values from the second column onwards in *G*. Consequently, the cost function can be represented as:

8 $$\begin{aligned} J(k) = U(k)^{ T} H U(k) + 2 x(k)^{ T} F^T U(k) + x(k)^{ T} V x_k \end{aligned}$$

where $H = G^T Q G + R$, $F = G^T Q W$, and $V = W^T Q W + Q$. The optimal input sequence $U(k)^*$ is obtained by solving the optimization problem in (9) as follows

9 $$\begin{aligned} \begin{aligned} U(k)^*&= \underset{U(k)}{argmin}\ \quad U(k)^T H U(k) + 2 X(k)^T F^T U(k)\\&\text {s.t.} \quad A_{cs} U(k) \le b_{cs} + B_{cs} X(k) \end{aligned} \end{aligned}$$

where $b_{cs} = [X_{max}, - X_{min}]^T$, $B_{cs} = [-A^i, A^i]^T$, and $A_{cs} = [G_i, -G_i]^T$. The inequalities in (9) represent the constraints and are handled using the active set method and Karush-Kuhn-Tucker conditions. The diagram of applying linear MPC is shown in Fig. 3(b).

### Uncertainty analysis and sensitivity analysis

We employ uncertainty analysis and sensitivity analysis to elucidate the varying patient responses to therapy, shedding light on how parameter perturbations can reveal the impact of inherent variability in individuals undergoing cancer treatments.

Uncertainty analysis of the model is conducted to assess the robustness when faced with parameter uncertainty arising from limited data and patient variability. For model parameter uncertainty analysis, the model can be denoted as $Y = f_{u}(P_1, P_2, \ldots , P_n)$, relates the model output (*Y*) to a set of model parameters ($P_i$), where *n* represents the number of parameters, and $f_{u}$ represents the function relating input parameters to the output. It is assumed that the model parameters follow normal distributions, expressed as $P_i \sim {\mathcal {N}}(\mu _i, \sigma _i^{2})$, where $\mu _i$ signifies the nominal value of $P_i$, and a standard deviation of $\sigma _i = 0.1 \mu _i$ is employed. For analysis of uncertain initial conditions, the potential inaccuracies in the initial values of the four states are caused by noisy measurements in imaging methods. For instance, immunohistochemistry has been reported to achieve up to 90% accuracy^49^. Thus, we assume a uniform distribution for the measured noisy initial conditions to address this. Specifically, if the measured initial state is denoted as *X*(0), the true initial conditions are assumed to follow a uniform distribution $U[\frac{X(0)}{1.1}, \frac{X(0)}{0.9}]$.

Variance-based sensitivity analysis is performed to assess parameter influence on the settling time of the controlled system using Sobol indices^50^. A model $Y_s = f_s(\varvec{P,x_0})$ has *n* changing parameters $\varvec{P} = \{par_1 , par_2, \ldots , par_n\}$. The model output is decomposed into $f_{s_i}(par_i)$, $f_{s_{ij}}(par_i, par_j)$, and so on, with corresponding variances $V_i$, $V_{ij}$, and higher-order terms. First-order indices $S_i$ and total-order indices $S_{T_i}$ are calculated to evaluate parameter contributions, using Python package SAlib^51,52^.

## Results

**Figure 4:**
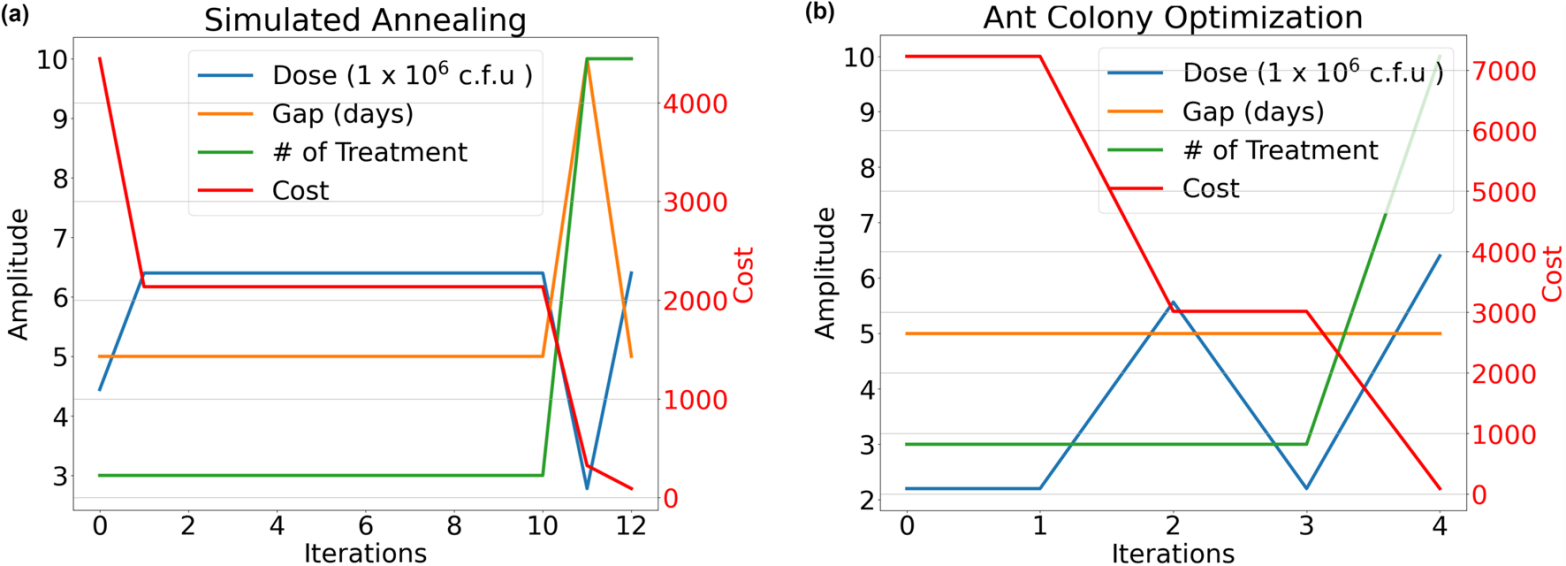
The best solutions and the minimal cost obtained by using (**a**) the Simulated Annealing method and (**b**) the Ant Colony Optimization. They obtain the same optimal solution $[d,g,N] = [6.4, 5, 10]$ with the lowest cost of 91.2. Both of the optimization techniques tend to obtain solutions with lower doses and more treatments for the settings used in this study.

RMC and Koopman MPC were used to design the optimal dosing schemes for BCG treatment. The initial state [0.1, 0.1, 0, 0.8] was used for testing for both designs, indicating an early-stage bladder cancer with few effector *T* cells (i.e., $E(0) = 0.1 \times 10^6$) and no infected tumor cells to begin with. The weights $w_1 = 200$, $\lambda = 1$, $w_N = 10^4$ and $w_t = 10^5$ were used in the cost function in Eq. (3). Also, the values of *B*(0), *E*(0), and $T_i(0)$ were relatively low compared to $T_u(0)$ to emphasize the decrease in $T_u$. Once determined, the drug doses and the gap between treatments remained unchanged during the simulation. The three optimizers started with the same initial condition $[d_0, g_0, N_0] = [2.2, 10, 3]$, i.e., the lowest dose, the largest gap, and the least number of treatments, indicating the dosing scheme was initiated at the least amount of doses that might not be sufficient to eliminate cancerous cells. In PSO, we used $w = c_1 = c_2 = 0.5$ and the swarm size of 100 particles and obtained the best solution $[d,g,N] = [5.46, 5, 10]$ with a cost of 87.9 in one iteration. In Simulated Annealing, we set $C_r = 0.95$ and initial temperature 1, and the best solution obtained was $[d,g,N] = [6.4, 5, 10]$ with a cost of 91.2, as shown in Fig. 4(a). In ACO, we used heuristics *G*, 1/*D*, and 1/*N* for priori information. An optimal solution $[d,g,N] = [6.4, 5, 10]$ with cost 91.2 was obtained, as shown in Fig. 4(b). Notably, this solution aligns with the best outcome obtained through simulated annealing. The results from three optimization algorithms showed that PSO quickly converged to an optimal solution in a single iteration, while ACO and Simulated Annealing required more iterations. PSO outperformed the others with the lowest-cost solution. Note that tuning parameters in the algorithms can potentially improve performance further and reduce costs.

Additionally, the optimal combination of *g* and *N* touched the boundaries of the constraints. This validates our design, as *d* and *N* tended to increase and *g* tended to decrease to obtain a more aggressive control action to eliminate cancerous cells. To verify if the solution is a global optimum or a local optimum, we expanded the variable boundaries for *d* ([2.2, 6.4] to [0, 50]), *g* ([5, 10] to [1, 20]), and *N* ([3, 10] to [1, 20]), which improved the solution to [3.22, 1, 20] with a lower cost of 82.2 after 1 iteration (by using same setting in PSO), in contrast to the original boundaries ($[d,g,N] = [5.46, 5, 10]$ with a cost of 87.9). This suggests that the initial solution was a local minimum. However, certain constraints remain crucial for clinical feasibility and FDA guideline adherence, especially regarding safety with BCG doses.

**Figure 5:**
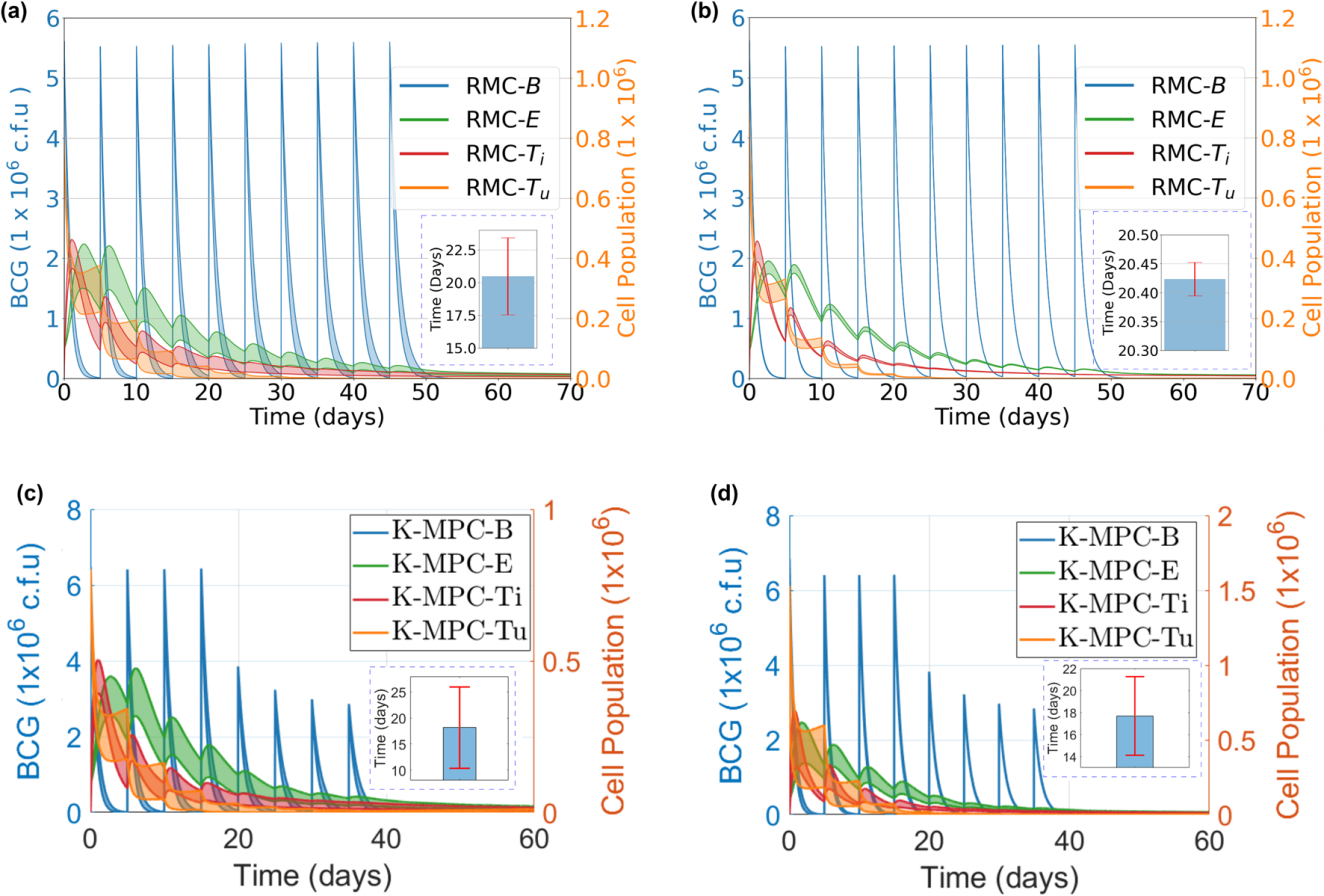
Uncertainty analysis with (**a**,**b**) RMC-derived inputs and (**c**,**d**) KMPC-derived inputs. The time that $T_u$ is brought to be below 1% of its initial value is used as the measurement. (**a**) Parameter uncertainty analysis shows an average time of 20.48 days with a standard deviation of 2.94 days. (**b**) Initial condition uncertainty analysis shows an average time of 20.42 days with a standard deviation of 0.03 days. (**c**) With inputs from KMPC, parameter uncertainty analysis shows an average time of 18.15 days with a standard deviation of 3.94 days. (**d**) With inputs from KMPC, initial condition uncertainty analysis shows an average time of 17.70 days with a standard deviation of 1.79 days. The shadow area represents the 95% confidence interval. The bar plots show the statistics of the time *t* for $T_u(t) < 0.01 T_u(0)$.

The analysis of uncertain model parameters entails 200 Monte Carlo runs using a 95% confidence level. The results for the uncertain model parameters and uncertain initial conditions using the dosing scheme by RMC are visually depicted in the shaded regions within Fig. 5(a) and (b), respectively. We used the time that $T_u$ was brought to be below 1% of its initial value as a reference to assess the performance. For models with uncertain parameters, we observed an average time of 20.48 days with a standard deviation of 2.94 days. For models with uncertain initial conditions, the average time was 20.42 days with a standard deviation of 0.03 days. Figure 5(c) and d show the results of uncertain model parameters and uncertain initial conditions for KMPC, which has an average time of 18.15 days with a standard deviation of 3.94 days, and an average time of 17.70 days with a standard deviation of 1.79 days, respectively.

**Figure 6:**
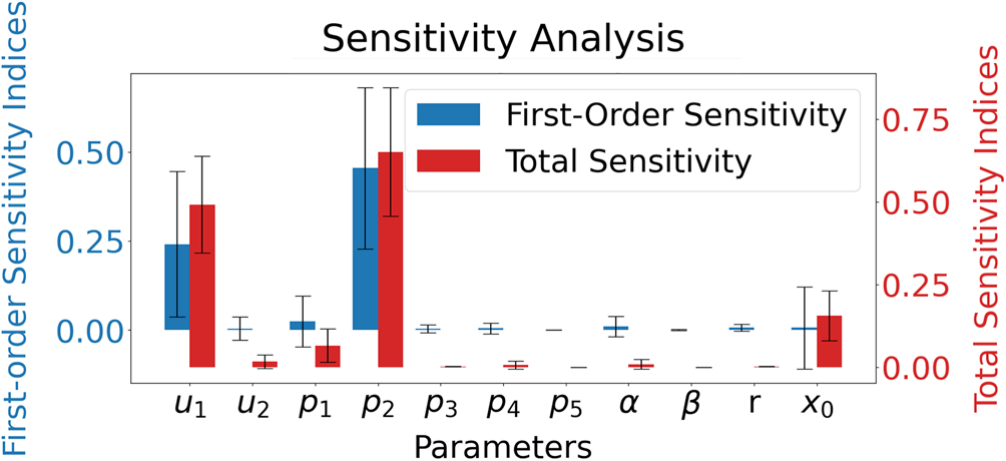
Sobol sensitivity analysis on model parameters. The first-order and the total-order Sobol indices are visualized, and the higher-order indices are ignored. The output settling time is sensitive to the changes in the parameters $p_2$ and $\mu_1$.

In sensitivity analysis, we considered parameters in Table 1, assuming uniform distributions spanning 0.8 to 1.2 times their nominal values. The initial state $x_0$ was treated as a unified entity and only different scales of the same $x_0$ were considered, simplifying the analysis while examining its influence. The output $Y_u$ represents settling time. Figure 6 shows first-order and total-order Sobol indices from 1000 iterations. The parameter *p*_*4*_ has total and first-order Sobol indices of 0.0064 and 0.0046, in contrast to more impactful parameters like *p*_*2*_, with total and first-order Sobol indices of 0.65 and 0.45. This shows the output settling time is not sensitive to the selection of numerical values of *p*_*4*_, as claimed earlier in the model section.

**Figure 7:**
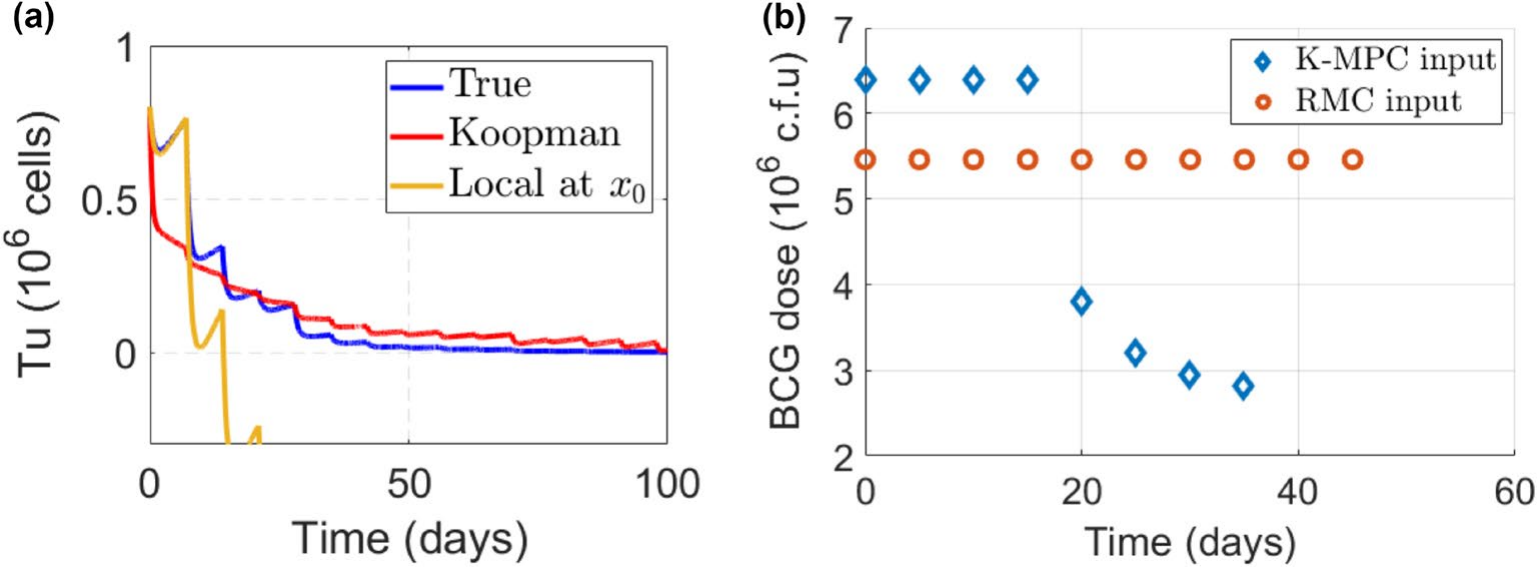
(**a**) The Koopman operator demonstrates effective linearization performance. Compared to the linear approximation (in yellow curve), Koopman linearization (in red curve) closely aligns the dynamics of the model observable with the output from the nonlinear model (in blue curve). (**b**) The comparison of the dosing schemes by RMC and Koopman MPC. RMC employs a gentle dosing scheme, resulting in a longer treatment duration. In contrast, Koopman MPC implements an aggressive control action with high doses administered for over half of the treatment period, leading to faster elimination of tumor cells.

For Koopman MPC, the linear Koopman operator was approximated using the spectral method with 100 trajectories of time series data (generated from the nominal model with random initial conditions and inputs). Ten RBFs were tested with good performance and their centers were obtained by the K-means algorithm. The sampling time of 0.01 days was used, and the simulation length was 100 days. The delay of 1 was used for one-step time delay embedding. The control interval was set to 5 days, which is the same as the treatment interval in RMC for comparison. The accuracy of linearization was evaluated by comparing the model output (i.e., $T_u$) as shown in Fig. 7(a). The output of the Koopman-based linear model (in red) is closer to the model dynamic behavior (in yellow) for most of the time during the simulation, compared to local linearization using the truncated Taylor expansion, even though it had more deviation from the true dynamic behaviors at the beginning, indicating its good approximation performance over time. Linear MPC was applied to the Koopman-linearized model to optimize BCG dosing. The simulation involved the administration of BCG vaccines every 5 days, and a prediction horizon of 5 days was used to balance control performance and computational complexity. The control horizon was set to one sampling time. Weighting matrices $Q = diag(0, 0, 0, 1000)$ and $R = 0.1$ were used to focus more on state errors.

Both dosing schemes effectively eliminated the uninfected tumor cells but differed in their treatment plans. Figure 7(b) compares the dosing schemes designed by RMC and Koopman MPC. For patients with identical initial conditions, RMC’s dosing scheme administered smaller doses during the initial phase of treatment, followed by larger doses in the later phase, in contrast to Koopman MPC’s approach. The aggressive control action in KMPC led to rapid elimination of cancerous cells, resulting in treatment completion in approximately 30 days. However, this approach may simultaneously induce more severe side effects, making it more suitable for patients who exhibit resistance or tolerance to high drug doses. On the other hand, the more gentle treatment plan illustrated in RMC resulted in a gradual reduction of tumor cells and a longer treatment duration.

## Conclusion and discussion

In this study, we introduced RMC as a novel control strategy for designing optimal drug dosing schemes in the context of a BCG treatment model for bladder cancer, and compared its performance to the existing Koopman MPC approach. Both strategies effectively eliminated uninfected tumor cells but differed in their underlying principles. RMC optimized the impulsive BCG dosing scheme by balancing cumulative doses and cancerous cell populations. Using PSO, we found the optimal solution for the variables that defined the dosing scheme. We compare the modeling results of using RMC with data from clinical trials on the BCG efficacy in^53^ and the side effects in^54^ from 1316 patients. These studies primarily employed the OncoTICE strain, characterized by containing 5 $\times 10^8$ colony-forming units (c.f.u) per standard dose. Given that approximately 99% of the BCG departs from the bladder within 2 hours post-instillation^28^, a dose of 5.6 $\times 10^6$ c.f.u of BCG, intended to be sustained within the bladder. The best dosing scheme in our study is $[D, G, N] = [5.46, 5, 10]$. Then the dose from our solution is roughly equivalent to 0.975 standard doses, and the 5-day gap in our proposed dosing scheme signifies a more intensive instillation schedule compared to the typical weekly BCG instillations. In total 10 treatments result in a cumulative treatment duration of 50 days, which is close to the standard course consisting of 6 weekly treatments. Note that the maintenance treatments are not considered here. Both studies concluded that low-dose treatment (i.e., 1/3 of the standard dose) was associated with a higher recurrence rate compared to the standard dose. Nevertheless, no significant differences in observed toxicity were reported across the varying dosage levels. Given that our dosing scheme approximates the standard doses, it can be inferred that the recurrence rate is likely to remain low. It’s worth noting that even a slightly shorter gap between instillations (from 7 to 5 days) could potentially yield different side effects. Consequently, evaluating potential side effects necessitates more extensive clinical trials for conclusive insights. Additionally, constraint relaxation is used to transform the hard constraints into soft constraints in the cost function. Exploring solutions that adhere to the hard constraints can also be achieved through mixed-integer optimization. For example, we can examine optimal solutions for *d* and *g* while setting *N* to values such as $N = \{5, 6, 7, 8, 9, 10\}$ to satisfy $N \in {\mathbb {Z}}^+$.

Koopman MPC leveraged the linearized model based on the Koopman operator, which closely resembles the original nonlinear dynamics of the uninfected tumor cell population. This was in contrast to the Taylor approximation, which required operating points and might not always be available. The control performance exhibited a desired decrease in the uninfected tumor cell population with the impulsive drug dosing regimen employed by both controllers. The optimality of these strategies effectively balanced the therapeutic performance of the treatment with the physical constraints of the patients. Considering that most disease and treatment models in the real world are nonlinear and many drugs are administered impulsively, the Koopman-based impulsive MPC framework holds promise for application in various disease treatments due to its linearity and impulsivity. The Koopman linearized model offers improved accuracy compared to the linear approximation, as it does not rely on specific operating points for its formulation.

The two dosing schemes have relatively gentle and aggressive doses, as shown in the above. The main reason for RMC to provide gentle control inputs is that we kept the dose *d* constant throughout the entire time horizon. Conversely, KMPC exhibited more aggressive dosing due to the absence of a penalty on the $\Delta u = u(t+1) - u(t)$ term, which restricts input increments in the cost function. To encourage a gentler dosing strategy akin to RMC, we can introduce the term $||\Delta u||_2^2$ weighted by $w_u$, where $||\cdot||_2$ denotes the *L*2 norm. Besides, one notable advantage of RMC over Koopman MPC is its ability to handle nonlinear models without the need for linearization techniques directly. This feature facilitates the generalization of RMC to other treatment models with impulsive drug doses and constraints. For practical consideration, RMC is cost-effective for healthcare settings with limited patient data, as it operates in an open-loop manner without requiring extensive patient measurements. In contrast, KMPC, which relies on patient feedback and measurement updates to maintain model accuracy, is more expensive but excels in adapting to significant changes in a patient’s condition. Note that KMPC is adapted to large changes in patient condition, as the uncertainty analysis already showed its robustness to small changes in model parameters. The choice between RMC and KMPC depends on the specific resource and adaptability requirements of the healthcare context. Additionally, in this study, the magnitude and gap of doses remained unchanged throughout the entire treatment. However, there is potential for further improvement by allowing these values to vary, potentially leading to lower values of the objective function. This flexibility would enable customization of the dosing scheme for each treatment, considering the specific needs of patients.

The integration of abundant molecular data from cancer patients offers great promise for advancing precision medicine. One potential approach involves identifying parameters influenced by gene expression variations related to cancer and treatment, then quantifying these relationships. For instance, genes like *BCL2* and *BAX*, known apoptosis regulators^55^, may impact the cancer cell growth rate *r*. Similarly, the gene *CD*4 could contribute to the activation rate of the immune response $$p_4$$ due to BCG treatment-induced effects on effector T cells $$CD4^+$$^56^. Recent research analyzing genomic profiles in bladder cancer patients receiving BCG treatment has identified differentially expressed genes and signaling pathways^57^, potentially establishing quantitative links between gene expressions and model parameters. These connections could be clarified by linking parameter functions with relevant signaling pathways.

Overall, the reparameterization of the control process effectively addresses the nonlinearity of the model and enables the inclusion of multiple control objectives, including constrained optimization. The proposed control scheme has the potential for generalization to similar control problems with minimal modifications, streamlining the control design process and enhancing scalability.

## Data Availability

The code and data that support the findings of this study are available from the corresponding author, Yue, R, upon reasonable request.
